# Ginger Extract and Fulvic Acid Foliar Applications as Novel Practical Approaches to Improve the Growth and Productivity of Damask Rose

**DOI:** 10.3390/plants11030412

**Published:** 2022-02-02

**Authors:** Esmat F. Ali, Hatim M. Al-Yasi, Ahmed A. Issa, Kamel Hessini, Fahmy A. S. Hassan

**Affiliations:** 1 Department of Biology, College of Science, Taif University, Taif 21944, Saudi Arabia; h.alyasi@tu.edu.sa (H.M.A.-Y.); a.hissa@tu.edu.sa (A.A.I.); k.youssef@tu.edu.sa (K.H.); 2 Horticulture Department, Faculty of Agriculture, Tanta University, Tanta 31527, Egypt; fahmy_hssn@yahoo.com

**Keywords:** biostimulants, nutrient elements, anthocyanins, phenolics, carotenoids, volatile oil

## Abstract

Plant biostimulants (BIOs) have been identified as among the best agricultural practices over the past few decades. Ginger extract (GE) and fulvic acid (FA) are a new family of multifunctional BIOs that positively affect development processes in plants. However, the underlying mechanisms that influence these development processes are still unknown. The objective of this study was to determine how GE and FA affect the plant growth and productivity in damask rose. Furthermore, the mechanisms of these BIOs that regulate the performance of this plant were investigated. Damask rose plants were foliar-sprayed with GE (5, 10 and 15 mg L^−1^) or FA (1, 3 and 5 g L^−1^), while control plants were sprayed with tap water. The results showed that GE or FA foliar applications enhanced plant height and branch number much more than the control; however, FA treatment was more effective than GE. Intriguingly, flower number, flower yield, relative water content, and total chlorophyll content were all improved by either GE or FA, paying attention to reducing the blind shoot number per plant. Relative to the control, foliar application with 15 mg L^−1^ GE or 3 mg L^−1^FA increased the flower number by 16.11% and 19.83% and the flower yield per hectare by 40.53% and 52.75%, respectively. Substantial enhancements in volatile oil content and oil yield were observed due to GE and FA treatments, especially with the highest concentrations of both BIOs. The treatments of GE and FA considerably improved the total soluble sugars, total phenolic content, total anthocyanin content, and total carotenoid content, more so with FA. Additionally, the contents of N, P, K, Mg, Fe, and Zn elements were also enhanced by applying either GE or FA, especially at higher levels of both BIOs. In sum, our findings illuminate the potential functions of exogenous application of GE and FA in improving the growth, flower yield, and volatile oil yield in damask rose through enhancing the phytochemical and nutrient profiles. Applications of GE and FA can, thus, be a promising approach for enhancing the productivity of damask rose.

## 1. Introduction

*Rosa damascena* Mill. (Damask rose) is a significant fragrant plant in the Rosaceae family that is widely utilized as a decorative species in gardens and parks, as well as in the perfume, cosmetic, and pharmaceutical industries [1]. The damask rose is the most important of the more than 200 Rosa genera found in Europe, North America, Asia, and the Middle East [2,3]. Damask rose flowers contain a volatile oil with antibacterial, anti-diabetic, anti-inflammatory, antioxidant, and antiviral activities [4]. The soil in dry and semi-arid locations is usually alkaline and has limited organic matter, which lowers macro- and micronutrient solubility and mobility [5]. Chemical fertilizers have been widely utilized to solve this problem; however, along with their expensive cost, their long-term usage has resulted in a number of concerns, including the destruction of soil microbial communities and the contamination of the environment [6,7]. Therefore, developing sustainable protocols to improve the growth and productivity of damask rose has spurred a massive interest in commercial applications to reduce the use of chemical fertilizers. Therefore, it is appropriate to search for safe alternatives to these chemicals, which can play a key role in increasing production and improving quality, and among these alternatives are BIOs, which are in high demand for the purposes of organic agriculture and sustainable production at a relatively low cost [8]. Recently, the use of BIOs has dramatically grown in agriculture because they improve the plant growth when used in low levels and improve the nutrient uptake [9,10,11,12]. It has been reported that BIOs are able to improve the productivity of some aromatic species [8,9].

*Zingiber officinale* Roscoe (Ginger) rhizomes, which belong to the Zingiberaceae family, are widely used as a spice, medicinal supplement, and dietary supplement [13]. Ginger extract (GE) of roots contains compounds (6-gingerol and its derivatives) with high antioxidant activity [14]. The major components of ginger are zingiberene, camphene, sabinene, α-farnesene, β-sesquiphellandrene, α-curcumene, geranial, and neral [13]. Additionally, curcumin, which is detected in ginger, considerably attenuated malathion-induced lipid peroxidation and oxidative stress [15]. GE also contains several antioxidants, such as flavonoids, phenolic acids (gallic, cinnamic, salicylic, vanillic, ferulic, and tannic acids), and ascorbic acid [16,17,18,19]. GE has been used as a natural biostimulant [20], and its foliar application improved the growth, total soluble sugar, volatile oil content, and nitrogen content of *Origanum majorana* [21]. Therefore, it is expected to be an effective biostimulant and growth promoter as an alternative to chemical fertilizers. Unfortunately, information about how GE can regulate the volatile oil production and enhance the phytochemical profile in damask rose is yet unavailable.

The use of plant BIOs in the form of humic substances (HS) is an alternate technique for improving crop yield and soil fertility [6,22,23,24,25]. HS are the most important sources of organic matter derived from plant and animal residues [26]. The use of HS is an important strategy to reduce the use of agrochemicals and fertilizers, improve soil conditions, and increase nutrient uptake by plants [27,28,29,30]. HS have been shown to have impacts on plant growth, yield, nutrient uptake, and access to metals [31,32,33,34,35]. Mineral transport is improved, protein synthesis is improved, enzyme activities are modified, photosynthesis is promoted, and micro- and macro-element solubility are increased due to HS [36,37]. Fulvic acid (FA) has a lower molecular weight, more acidic groups, and a higher oxygen content [38] and has been reported to be more effective as a foliar than humic acid [39]. FA has a higher efficiency as a foliar spray compared to humic acid, due to its higher solubility in low pH media, which is typical of foliar spray [40]. Additionally, FA has a greater efficacy as a foliar spray than as a soil amendment [41]; therefore, FA was chosen as a foliar spray in the current investigation. 

FA, the second most important humus substance, is one of the main potential BIOs [10] that improves forage crop production [42,43]. FA, as a biostimulant, is a non-toxic water binder, which maximizes the uptake via leaves and stimulates plant productivity. Furthermore, it attracts water molecules, relieves the nutrients’ movement into the roots, and easily binds or chelates minerals [44]. Foliar application of FA can be an eco-friendly and effective approach for attaining agronomic bio-fortification [45]. The most significant biological effects of FA include the facilitation of mineral nutrient uptake, improvement of growth, stimulation of biomass accumulation, and induced plant resistance to environmental stresses [10,28,46]. Application of FA enhanced the growth parameters, photosynthetic pigments, total phenols, total flavonoids, and antioxidants of yarrow [47,48]. FA treatment improved the growth characteristics and yield attributes of wheat [49] and sweet pepper [50]. Additionally, a significant increase in plant height, herb dry weight, leaf area, leaf number, chlorophyll content, and the contents of N, P, and K were observed due to FA foliar application over the control [51]. Application of FA increased the yield and nutrient uptake in gerbera [52] and pepper [53] and also increased carbohydrate, carotenoids, and total phenols in pepper [54].

Despite the impact of FA on improving the growth parameters and yield in several species, its mode of action remains unclear [41,43]. Furthermore, based on a review of the current literature, no published reports on the impacts of GE or FA on damask rose growth and productivity are available. Therefore, an in-depth study of its response to GE and FA treatments and the mechanisms involved may help to identify how to enhance the growth and productivity of damask rose. Hence, this experiment aimed to investigate the impact of GE and FA on the growth characteristics, flower yield, and volatile oil yield attributes of damask rose. Additionally, the underlying mechanisms that might be involved in its growth promotion were investigated through some physiological and phytochemical assessments.

## 2. Material and Methods

### 2.1. Experimental Procedure and Location

This field experiment was conducted at a private farm, located in a highland region (1700 m altitude, 21°26′02.4″ N 40°29′36.9″ E) of the Taif Governorate, Saudi Arabia, through the 2021 growing season, to study the effects of foliar spray with GE and FA concentrations on the growth and productivity of damask rose (*Rosa damascena* Mill. var. *trigentipetala*). Seven-year-old uniform shrubs cultivated at 2 m within and between the rows (2500 hill/ha) were chosen to perform the experiment. This experiment had seven treatments, each with four replicates, and it was laid out as a completely randomized design (CRD). The shrubs were pruned at 80 cm from the ground level on 1 January. A fixed dose of decomposed organic fertilizer (3 kg/hill ) was supplied immediately after pruning. The other cultural practices (irrigation, weed control, insect and disease control, etc.) were performed when required. One month later (with shoot appearance after pruning), foliar sprays with ginger extract at 5, 10, and 15 mg L^−1^and fulvic acid at 1, 3, and 5 g L^−1^ were applied four times in 14-day intervals. Control plants were foliar-sprayed using tap water. 

### 2.2. Preparation of the GE and FA Levels

The GE was performed as described by Shabana et al. [21], with slight modifications. Briefly, ginger rhizomes were air-dried and ground to obtain a fine powder. From this powder, 100 g was extracted by ethanol (80%), filtered 3 times by Whatman No. 1, and then evaporated to dryness under reduced pressure at 40 °C using a rotary evaporator. Finally, 3 concentrations of 5, 10, and 15 mg L^−1^ were used for foliar spray denoted as GE1, GE2, and GE3, respectively. The chemical analysis of the ginger extract is presented in Table 1. FA (C_14_H_12_O_8_, MW., 308.24) was obtained from Sigma Aldrich Co. (St. Louis, USA, CDS025195) and the concentrations of 1, 3, and 5 g L^−1^ were prepared using distilled water and used for foliar spray denoted as FA1, FA2, and FA3, respectively. 

For plant analysis, three composite samples (leaves and stems) of trimmed vegetative wastes were taken from each farm. Plant materials were rinsed in tap water, then distilled water, and air-dried at room temperature in the shade before being homogenized in a planetary high-energy mill with a hardened chromium steel vial.

### 2.3. Growth and Flower Yield Measurements

During the flowering stage, the flowers were continuously collected, and the flower yield for each hill was finally determined (kg/hill). The flower yield per hectare was also calculated using the number of hills (kg/ha), as well as the blind shoots per hill (branches that bear unopened flower buds or do not turn into flowers). In each hill, the plant height (cm) and the number of main new shoots were measured.

### 2.4. Volatile Oil Assessment and GC-MS Analysis

The volatile oil from flowers was extracted following a hydro-distillation method by a Clevenger-type apparatus according to the British Pharmacopea [55]. The percentage of volatile oil was calculated based on the sample fresh weight (FW) using the following formula:

Volatile oil (%) = oil volume/sample FW × 100(1)

The volatile oil yield (L ha^−1^) was then calculated based on the flower yield (kg ha^−1^), which was previously determined. The collected volatile oil was dehydrated using Na_2_SO_4_ and kept at 4 °C until GC-MS investigation. A Varian GC (CP-3800) and MS (Saturn 2200) equipped with a capillary column (VF-5 ms 30 × 0.25 mm ID and film thickness 0.25 µm) was used to investigate the volatile oil components. The energy of electron system ionization was 70 eV to detect the GC-MS. To identify the volatile oil components, the retention index (RI) of each peak was compared with standards and the GC-MS NIST library.

### 2.5. Relative Water Content (RWC)

The method described by Weatherley [56] was used to determine the leaf RWC by the following equation:

(S_FW_ – S_DW_)/(S_TW_ – S_DW_) × 100 (2)

where S_FW_, S_DW_, and S_TW_ are the sample fresh weight, dry weight (oven-dried at 70 °C for 48 h), and turgid weight (after saturation in distilled water for 24 h at 4 °C), respectively.

### 2.6. Chlorophyll Assessment

The methodology described by Lichtenthaler and Wellburn [57] was followed to determine the total chlorophyll content (TCC). Leaf samples (0.2 g) were extracted in 80% acetone and centrifuged at 12.000× *g* for 15 min. The absorbance of the supernatant was investigated at 662 and 645 nm. The TCC was calculated by the sum of chlorophyll a and chlorophyll b using the following equations:

Chl a = 11.75.*A*_662_ − 2.35.*A*_645_ (3)

Chl b = 18.61.*A*_645_ − 3.96.*A*_662_ (4)

where *A*_x_ represents the optical density at the subscripted wavelength.

### 2.7. Total Soluble Sugar (TSS) Determination

TSS was measured according to Shi et al. [58]. Leaf samples (0.2 g) were homogenized in 5 mL of ethanol (96%) and centrifuged at 3500× *g* for 10 min. Each 1 mL of the supernatant was reacted with 3 mL of the anthrone reagent (150 mg anthrone + 100 mL concentrated H_2_SO_4_). The mixture was then incubated for 10 min in a boiling water bath. The absorbance was finally investigated at 630 nm by a spectrophotometer (LKB-Novaspec II, Pharmacia, Uppsala, Sweden) and TSS was measured by the standard of glucose and expressed as milligrams per gram of FW.

### 2.8. Estimation of Macro- and Micro-Elements

Leaf samples from the middle parts of shoots were collected and oven-dried at 70 °C for 48 h. After that, a sample of 0.5 g of fine powder of dried leaf samples was digested using the mixture of HClO_4_ and H_2_SO_4_ (1:5), as reported by the Association of Official Analytical Chemists (A.O.A.C.) [59]. The micro-Kjeldahl method was used to determine the N content, as described by Nelson and Sommers [60]. The P content was colorimetrically assessed by a spectrophotometer (LKB-Novaspec II, Pharmacia, Uppsala, Sweden), and the content of K was investigated by a flame photometer (Flame Photometer 410, Sherwood Scientific Ltd,1 The Paddocks, Cherry Hinton Road, Cambridge, CB1 8DH, United Kingdom), according to Prasad et al. [61]. The contents of Mg, Fe, and Zn were spectrophotometrically assessed, as described by A.O.A.C. [59].

### 2.9. Total Phenolic Content (TPC) Investigation

A 0.5 g sample of fine flower powder was put into a glass tube, then aqueous methanol (10 mL) was added, and the tube was kept in a water bath for 30 min at 80 °C and then cooled. The mixture was centrifuged for 30 min at 2150× *g* at 20 °C and the volume of collected supernatant was adjusted to 10 mL by aqueous methanol. The Folin–Ciocalteu reagent was used to investigate the TPC according to Kamtekar et al. [62], using gallic acid as a standard. The absorbance was measured at 750 nm using a spectrophotometer (LKB-Novaspec II, Pharmacia, Uppsala, Sweden). The values were expressed as milligrams of gallic acid equivalents (GAE) per gram of sample dry weight (mg GAE/g DW).

### 2.10. Total Anthocyanin Content (TAC)

The method described by Wrolstad et al. [63] and modified by Hnin et al. [64] was used to determine the TAC. Each 0.3 g flower sample was put into a 100 mL beaker containing acidified methanol (15 mL) and kept for 4 h at room temperature. The solution was then filtered (Whatman No. 1) and the filtrate was investigated at 530 nm. The following equation was used to calculate the TAC:

TAC (mg 100 g^−1^ DW) = A × MW × DF × 100/(Ɛ × W) (5)

where A is the absorbance, MW is molecular weight of cyanidin-3-glucoside (449.2 g mol^−1^), DF is the dilution factor, Ɛ is the molar absorptivity (26,900, molar extinction coefficient (L mol^−1^· cm^−1^) for cyanidin-3-glucoside), and W is the sample weight (g).

### 2.11. Total Carotenoids (TCs) Estimation

TCC was estimated in flowers, as described by Dóka et al. [65]. Each 5 mg of dried fine powder was extracted in 1 mL of acetonitrile (ACN):methanol (MeOH):tetrahydrofuran (THF) by 50:45:5 (*v*/*v*) in an Eppendorf, followed by shaking at 150 rpm for 2 h. Then, the solution was cold centrifuged (−5 °C) for 5 min at 8163.1× *g*. The filtered supernatant was spectrophotometrically assessed at 450 nm to determine the TCC. The sample and the standard, diluted in the same solvent mixture, were investigated and the TCC content was expressed on a β-carotene basis. 

### 2.12. Statistical Analysis

The results were subjected to an analysis of variance (ANOVA) in SPSS v 13.3 (IBM, Armonck, USA). The statistical differences were investigated by Duncan’s multiple range test at *p* ≤ 0.05 [66]. The values were recorded as means ± SE (*n* = 4). A principal component analysis (PCA) was performed using Analyse-it Software (v. 5.6 for Excel).

## 3. Results

### 3.1. Growth Characteristics

**Figure 1:**
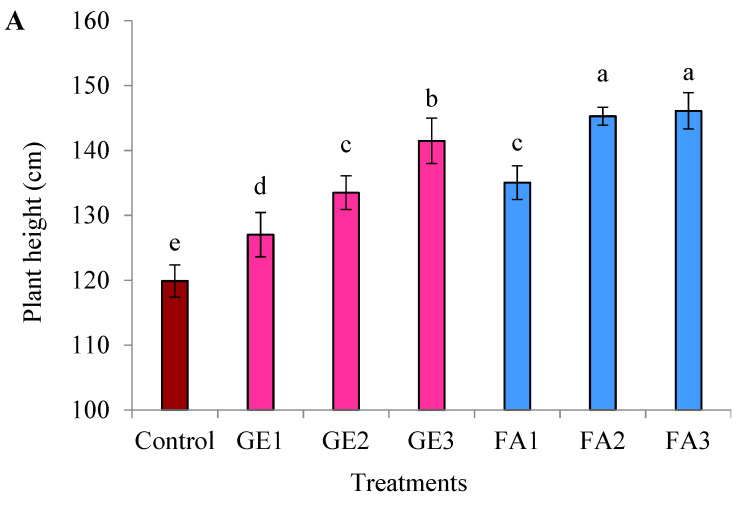

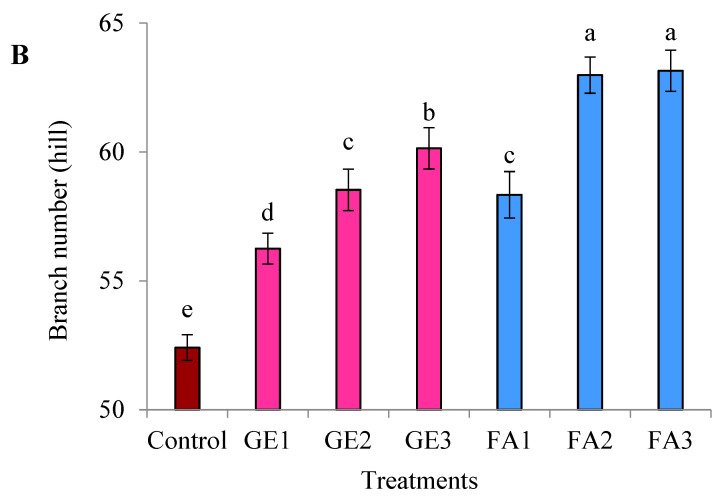
Effects of foliar spray with ginger extract and fulvic acid treatments on (**A**) the plant height and (**B**) the branch number of damask rose plants. GE1, GE2, and GE3 refer to ginger extract levels at 5, 10, and 15 mg L^−1^, while FA1, FA2, and FA3 are fulvic acid concentrations of 1, 3, and 5 g L^−1^, respectively. The data are means ± SE (*n* = 4). The columns with different letters are significantly different at *p* ≤ 0.05, according to Duncan’s multiple range test.

The plant height and branch number were significantly increased as a result of GE and FA applications compared to the control; however, FA treatment was more effective than GE (Figure 1A,B). Increasing the GE level gradually increased both characteristics, and they reached their maximum values when the highest level was applied (15 mg L^−1^). The same trend was recorded by FA treatment, but without significant differences between the 3 and 5 mg L^−1^ treatments.

### 3.2. Flower Yield Attributes

It is evident from the data in Table 2 that plants that were foliar-sprayed with GE and FA produced significantly higher flower number per hill, flower yield per hill, and flower yield per hectare in comparison to the control. However, GE and FA treatments significantly decreased the blind shoot number relative to untreated plants. Relative to the control, the flower number and flower yield per hectare were increased by 16.11% and 19.83% after applying 15 mg L^−1^ GE and by 40.53% and 52.75% after applying 5 mg L^−1^ FA, respectively.

### 3.3. Volatile Oil Content and GC-MS Analysis

**Figure 2:**
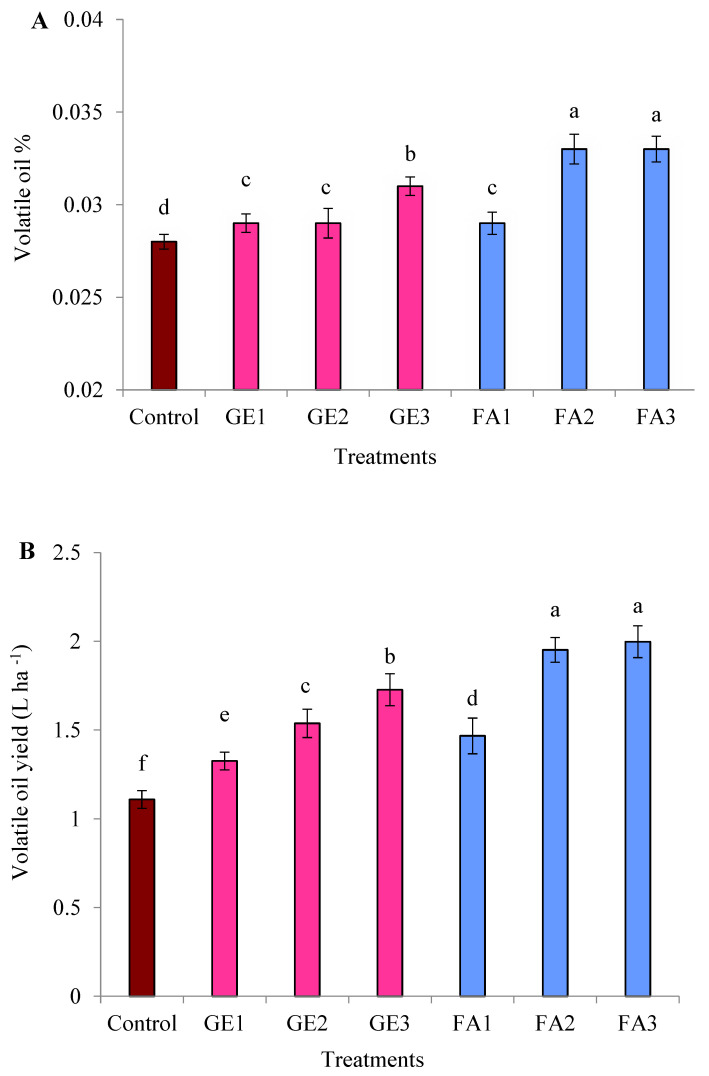
Effects of foliar spray with ginger extract and fulvic acid treatments on (**A**) the volatile oil percentage and (**B**) the volatile oil yield of damask rose plants. GE1, GE2, and GE3 are ginger extract levels at 5, 10, and 15 mg L^−1^, respectively; FA1, FA2, and FA3 are fulvic acid concentrations of 1, 3, and 5 g L^−1^, respectively. The data are means ± SE (*n* = 4). The columns with different letters are significantly different at *p* ≤ 0.05, according to Duncan’s multiple range test.

The volatile oil percentage and oil yield per hectare were significantly enhanced due to GE and FA treatments compared to the control. Generally, plants foliar-sprayed with FA produced higher volatile oil content than those treated with GE (Figure 2A,B). Among the treatments, the highest volatile oil percentage and yield per hectare were recorded with 5 mg L^−1^ FA without a significant difference from FA at 3 mg L^−1^. Applying GE at 15 mg L^−1^ increased the volatile oil percentage and yield per hectare by 10.71% and 55.69%, respectively, while these values were 17.86% and 80.02% when FA at 5 mg L^−1^ was applied. The GC-MS analysis of rose volatile oil shows that the main components detected were citronellol and geraniol, followed by nerol, linalool, and nonadecane (Table 3). Generally, the moderate or higher levels of both GE and FA improved the percentage of volatile oil components compared to the control.

### 3.4. Relative Water Content (RWC)

**Figure 3:**
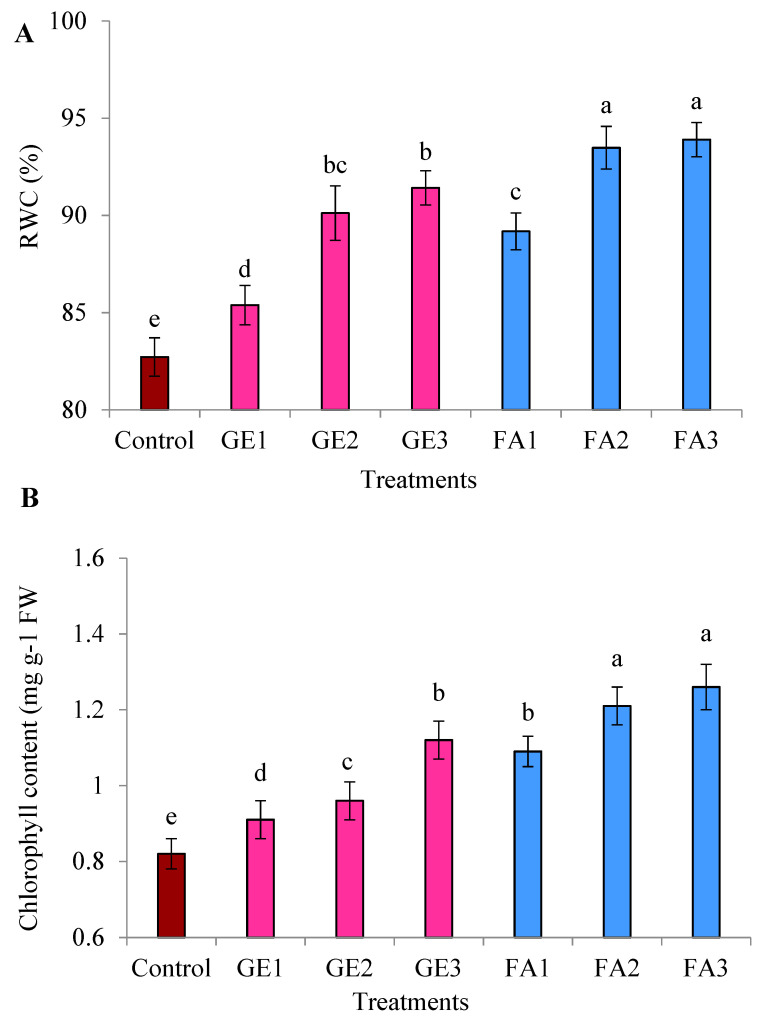

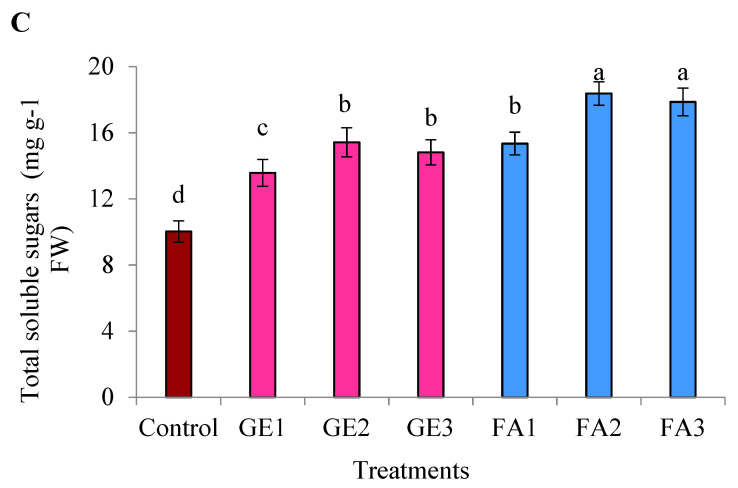
Effects of foliar spray with ginger extract and fulvic acid treatments on the (**A**) relative water content, (**B**) chlorophyll content, and (**C**) total soluble sugars of damask rose plants. GE1, GE2, and GE3 are ginger extract levels at 5, 10, and 15 mg L^−1^, respectively; FA1, FA2, and FA3 are fulvic acid concentrations of 1, 3, and 5 g L^−1^, respectively. The data are presented as means ± SE (*n* = 4). The columns with different letters are significantly different at *p* ≤ 0.05, according to Duncan’s multiple range test.

All levels of GE and FA remarkably increased RWC compared to untreated control. This effect was larger for FA (Figure 3A). This increment was gradual, with increasing amounts of GE and FA, but applying the highest levels did not have a significant impact relative to the moderate level. 

### 3.5. Total Chlorophyll Content (TCC)

The positive effects of the GE and FA treatments on leaf TCC were clearly observed, as control plants recorded significantly (*p* ≤ 0.05) lower contents than in GE- and FA-treated plants. The application of FA recorded a significantly higher TCC than the GE treatment, without a significant difference between FA2 and FA3 (Figure 3B). Compared with controls, plants foliar-sprayed with GE3 had a 36.59% higher chlorophyll level; the chlorophyll level in the FA3-treated plants was 53.66% higher. 

### 3.6. Total Soluble Sugars (TSS)

The treatments of GE and FA considerably improved the TSS in rose leaves in comparison to the control, and higher values were obtained by FA compared to the GE treatments (Figure 3C). Using GE2 and FA2 resulted in the highest TSS, and no impact was observed by increasing the levels to GE3 and FA3. Among all treatments, foliar spray with FA2 recorded the highest value, as it increased the TSS by 83.33% compared to untreated plants. 

### 3.7. Macro- and Microelements

It is clear from the data in Table 4 that increasing GE and FA levels significantly increased the contents of N, P, K, Mg, Fe, and Zn elements in damask rose leaves. This effect was larger for FA application. The highest level of GE recorded higher contents of the investigated elements than the low or moderate levels. Except Zn contents, plants foliar-sprayed with FA3 had higher contents of the investigated elements, but without significant differences compared to FA2.

### 3.8. Total Phenolic Content (TPC)

**Figure 4:**
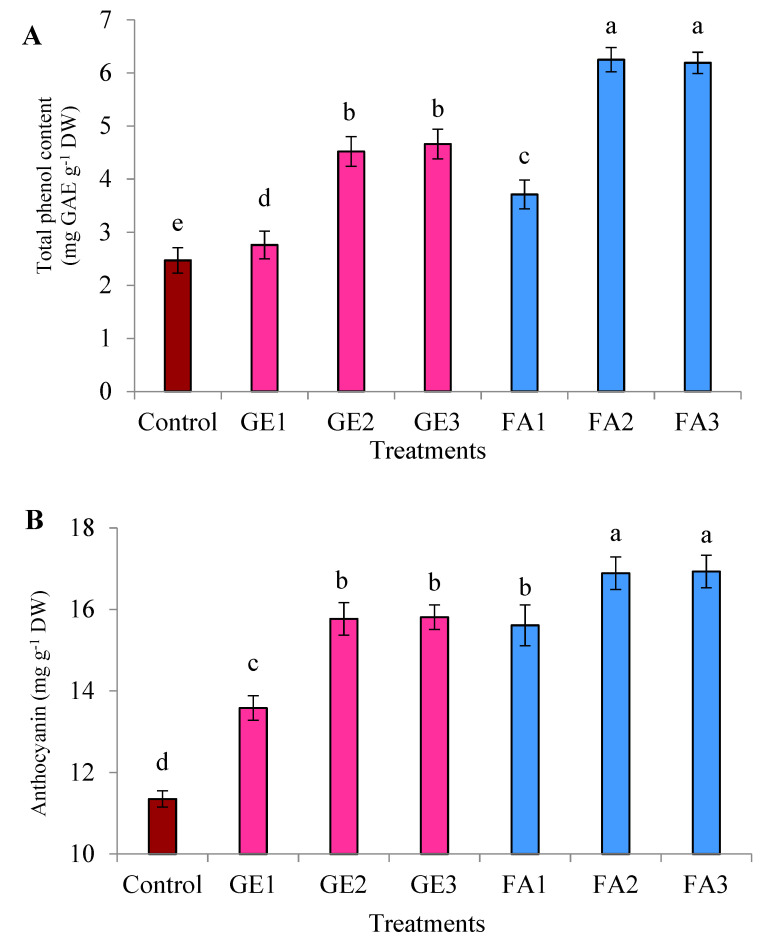

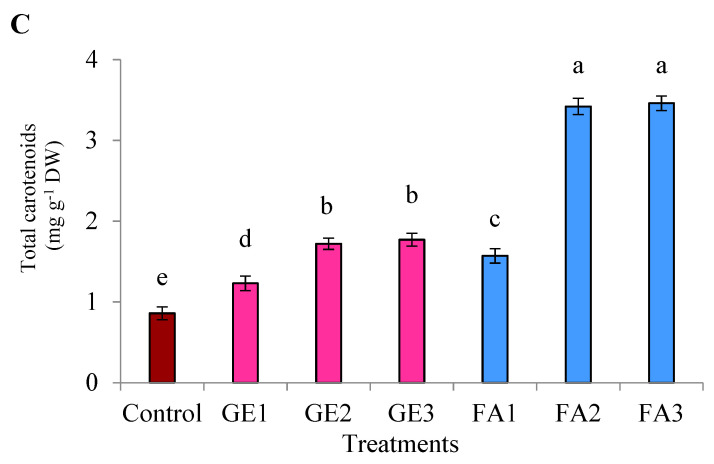
Effects of foliar spray with ginger extract and fulvic acid treatments on the (**A**) total phenol content, (**B**) anthocyanin content, and (**C**) total carotenoids of damask rose plants. GE1, GE2, and GE3 are ginger extract levels at 5, 10, and 15 mg L^−1^, respectively; FA1, FA2, and FA3 are fulvic acid concentrations at 1, 3, and 5 g L^−1^, respectively. The data are presented as means ± SE (*n* = 4). The columns with different letters are significantly different at *p* ≤ 0.05, according to Duncan’s multiple range test. DW: means dry weight.

The TPC was significantly increased due to GE and FA applications compared to the control. The moderate or higher levels of FA were more effective than any level of GA and recorded a higher TPC (Figure 4A). Applying GE3 slightly increased the TPC compared to GE2 without a significant difference. A slight decrease in the TPC was observed when increasing the FA level to FA3, but this reduction was not significant compared to FA2. The TPC was 2.53-fold higher than the control when the treatment of FA2 was applied.

### 3.9. Total Anthocyanin Content (TAC)

Foliar spray with GE and FA considerably enhanced the TAC in rose flowers compared to untreated plants, with a higher impact from FA (Figure 4B). Higher and moderate levels of GE recorded almost the same TAC. Applying FA3 slightly increased TAC relative to FA2, but the difference was not significant. Plants foliar-sprayed with FA2 had a 1.49-fold higher TAC than untreated plants.

### 3.10. Total Carotenoid (TCs)

The data presented in Figure 4C clearly show that TCs was significantly increased as a result of GE and FA foliar spraying compared to the control. This effect was larger with FA. Despite the increase in TCs observed with GE3 compared to GE2, the difference was not significant. Among the FA levels, FA2 recorded the highest TCs, and no impact was observed when FA3 was used, since it slightly increased the TCs. Relative to the control, foliar spray with FA3 increased the TCs by 4.02-fold.

### 3.11. Principal Component Analysis (PCA)

**Figure 5:**
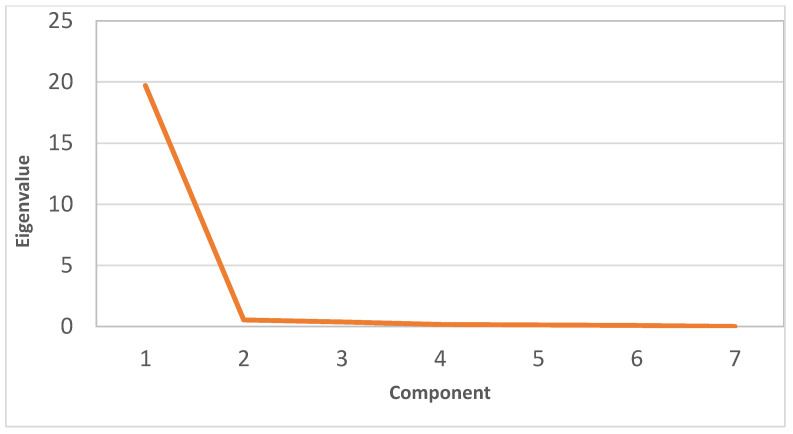
Scree plot of the PCA components for the damask rose parameters in response to the foliar application of ginger extract and fulvic acid.

**Figure 6:**
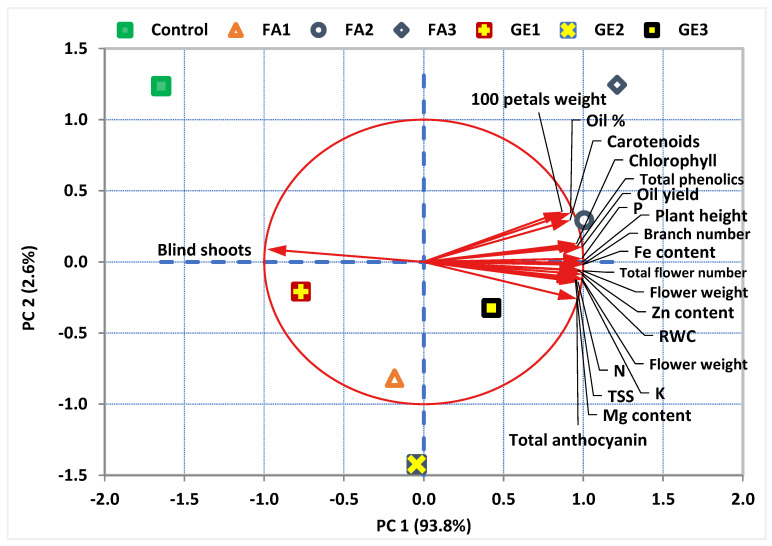
Biplot of the principal component analysis (PCA) for the damask rose parameters in response to the foliar application of ginger extract and fulvic acid. The first two principal components (PC1 and PC2) explain 96.4% of the total variation. The circle represents a perfect correlation. The vectors (arrows) represent the variables, and the colored shapes represent the sampling points under various treatments of fulvic acid (FA) and ginger extract (GE) in comparison to the control.

The principal component analysis (PCA) showed that only the first component had an eigenvalue greater than 1 and explained more than 93% of the variance in the data set, according to the scree plot (Figure 5) and the PCA biplot (Figure 6). PC1 and PC2 together explain 96.4% of the total variance. It appears that all the variables are positively correlated with PC1 except blind shoots, which showed a negative correlation. Meanwhile, the relation between the variables and PC2 varied. The biplot also provides invaluable information about the correlation between the variables. All variables showed a positive correlation with each other except blind shoots, which negatively correlated with all other variables. The positive correlation was weak between total anthocyanin and any carotenoids, oil %, or 100 petal weight. However, the positive correlation among the other variables was very strong. It is clear that tissue contents of Mg, N, K, and Zn had stronger relations with total anthocyanin than Fe and P content. On the contrary, P and Fe content had stronger relations with chlorophyll and carotenoids than Mg, N, K, and Zn. Both PC1 and PC2 successfully separated the effect of the applied treatments. Both the control and GE1 treatments seem to affect blind shoots. The FA2, FA3, and GE3 treatments are grouped together and seem to strongly affect all growth parameters.

## 4. Discussion

Developing eco-friendly materials to enhance the growth and productivity of damask rose has spurred a massive interest in commercial applications. In the current study, GE and FA were found to be effective BIOs to improve the growth, flower yield, and volatile oil content of damask rose. The current results indicate that GE is rich in carotenoids, carbohydrates, phenols, flavonoids, antioxidants, and macro- and micronutrients (Table 1). Due to these compounds detected in GE, it can be considered as a biostimulant to enhance damask rose growth and its productivity. The presence of such compounds in other extracts, such as moringa, improved the growth characteristics of geranium [9]. Enhancing damask rose growth with GE treatment supports the hypothesis of current study that GE can be an important plant growth enhancer. It is widely known that nutrient elements affect plant growth and development. Therefore, foliar spray with GE in the current study enhanced damask rose growth characteristics due to the presence of the nutrient elements required for better growth. In this context, rose plants maintained strong growth under adequate levels of nutrient elements [40]. Similarly, the growth attributes of rose plants were enhanced when the plants received proper levels of N, P, and K elements [67,68]. Furthermore, the P detected in GE is essential for root development promotion and, consequently, increased the water and nutrient absorption capability [69]. In agreement with our results, the effective role of several BIOs in improving the vegetative growth of different species has been reported [70,71].

Enhanced growth of damask rose plants in this study was also observed due to FA treatment. Application of FA enhances both root initiation and growth [72] and plays an outstanding role in cell signal transduction [6], having improved the vegetative growth of several species [41,73,74]. Additionally, FA might act as an organic carbon source and a stimulus to enhance cell growth and lipid accumulation [75]. The effective role of FA in increasing the uptake of nutrients, as our data indicated, may also explain the enhanced growth characteristics in treated plants, since a positive correlation was observed between the growth characteristics and nutrient elements (Figure 6). In the same vein, the application of humic substances improved plant growth by affecting cell metabolism and enhancing nutrient uptake [53,76]. Enhancing the growth by FA treatment may be also ascribed to the fact that FA contains plant signaling molecules or growth hormones such as an auxin-like molecule [77], that can alter plant metabolic processes [10,78]. Additionally, the enhanced growth with FA application may be also attributed to the role of FA in increasing the expression levels of mitogen-activated protein kinases and improving the protein content [75,79]. In agreement with the current results, the impact of FA on stimulating the growth of several species has been reported [39,48,76,80]. 

In the current study, foliar application with GE markedly increased the TCC, TSS, and RWC in damask rose leaves. A positive correlation among these parameters was observed (Figure 6). Enhancing the TCC may be ascribed to the Mg element, which is a chlorophyll constituent already found in GE (Table 1). One of the main impacts of BIOs is maintaining the leaf turgidity and enhancing the water relations, which results in retaining the leaf RWC and TCC [51,81]. Similar improvements in the TCC have been found with moringa leaf extract [9]. The increasing TSS in rose leaves by GE application noted here is in agreement with the report of Shabana et al. [21] in *Origanum majorana*. Improving both the TCC and TSS due to GE foliar application has been previously reported [71] in the common bean that has been foliar-sprayed with moringa extract. Similarly, FA treatment significantly maintained the RWC and enhanced both tte TCC and TSS relative to untreated plants. A higher RWC in treated leaves may be due to the effect of FA on improving the root system and enhancing water absorption [69]. In agreement with the current results, the role of FA in increasing the TCC and TSS has been documented in several previous reports [48,74,78]. 

Foliar spray with GE increased N, P, K, Mg, Fe, and Zn contents in rose leaves. This increase could be attributed to the fact that GE is rich in minerals, as shown in Table 1; therefore, its foliar spray increased the nutrient content in rose leaves. A similar trend was observed when plants were foliar-sprayed with other BIOs, such as moringa extract [8,82]. The high content of nutrient elements in GE makes it a suitable biostimulator that is able to increase the leaf absorption of essential nutrients. This is reflected in the improved growth characteristics indicated in our data. It is established that humic substances are important strategies to increase nutrient uptake by plants [10,25] through the induction of plasma membrane H^+^-ATPase activity [83] and by increasing the growth of roots in the rhizosphere [84]. Herein, FA application also increased the nutrient elements (N, P, K, Mg, Fe, and Zn) in damask rose leaves. Consistent with these results, previous reports have shown that FA can stimulate nutrient uptake by roots or foliage, and complex micronutrients are absorbed in a soluble form in diverse species [85,86]. It has been reported that FA application markedly enhanced the concentrations and bioavailability of N, Zn, and Fe elements [45]. Moreover, FA facilitates the absorption and translocation of elements that are involved in photosynthesis [19]. Providing more nutrients, particularly N, Mg, and Fe, due to FA treatment, enhances the biosynthesis of photosynthetic pigments and, therefore, improves the plant growth [48,83], which supports our results. Furthermore, a positive correlation between the nutrient elements and photosynthetic pigments was observed in the current study (Figure 5). Similar findings were observed by Yazdani et al. [76] in gerbera.

In this study, the flower yield attributes of damask rose were considerably enhanced as a result of GE application. Depending on the GE composition of several essential components required for better growth and flower production of damask rose, GE can be applied as a novel biostimulant for enhancing plant productivity, in line with our hypothesis [21]. GE may enhance the flower yield by improving nutrient acquisition and distribution within the plant and by improving both photosynthetic pigments and the TSS, which enhance plant growth and, consequently, flower yield. Furthermore, after a biostimulant’s entrance into the plant tissues, it controls the hormonal status during the growth and development and, therefore, increases plant productivity [81,87]. In agreement with our results, BIOs have been found to be excellent substances for improving not only the growth but also the yield of several crops [8,88,89,90]. In particular, FA application markedly improved the flower yield of damask rose. In agreement with our results, foliar application of FA improved the yield components of several crops [73,74]. Furthermore, the impact of FA on enhancing nutrient absorption and promoting plant growth may reflect in increasing the flower yield. Furthermore, a positive correlation between the growth characteristics and the flower yield was observed in the current study (Figure 5). It has been found that humic substances improve the yield attributes by stimulating nutrient absorption [52,91]. 

The current research is the first on damask rose to look at how volatile oil content reacts to the foliar application of GE. The obtained results reveal that GE is a promising biostimulant that positively influences the physiological and biochemical attributes that directly affect the terpenoid pathway, which may increase the oil content of damask rose. In the current investigation, GE treatment enhanced the volatile oil content and its composition. These findings may be attributed to the detected components in GE, particularly the essential elements, carbohydrates, and phenols that can encourage the accumulation of secondary metabolites. Moreover, the enhanced flower yield due to GE treatment may explain the observed increase in volatile oil yield. These results are in accordance with the report of Shabana et al. [21], who found that foliar application of GE led to quantitative and qualitative changes in the volatile oil constituents of *Origanium majorana*. Similarly, the impact of BIOs on enhancing the volatile oil yield and its composition has been found in several species [8,92]. The volatile oil content of damask rose was also improved by FA application. It has been reported that FA regulates hormone levels and contributes to the development of secondary metabolites [80]. In this regard, Santiago et al. [93] reported that FA enhanced the secondary metabolites by increasing and chelating nutrient uptake in saffron. The current results support the previous reports of Noroozisharaf and Kavian [94], who found that humic substances increased the volatile oil content and enhanced its major components in *Thymus vulgaris* L. 

Because of their health-promoting properties, phenolics have gained substantial attention due to their antioxidant activities [95]. The results in the current study showed that either GE or FA treatments improved the total phenolic content of damask rose flowers in comparison to control treatment. The higher phenolic content in GE-treated plants may be attributed to the higher content of phenolics and flavonoids detected in GE (Table 1) that might have contributed to the improved total phenolic content in damask rose flowers. A similar trend has been observed for other BIOs [8,96]. Furthermore, the appropriate concentration of minerals and vitamins observed in GE may directly or indirectly affect the metabolic processes in such a way that it enhances the internal phenolic content [19]. In agreement with current results, Shabana et al. [21] reported that the total phenols were improved due to a foliar application of GE in *Origanum majorana* L. Similarly, FA application enhanced the total phenols in damask rose flowers. It has been revealed that the compounds related to the shikimic pathway, such as phenols, are agitated by humic substances [97]. In addition, non-enzymatic antioxidants such as phenols and carotenoids have been influenced by the application of humic substances [6]. In the same context, Bayat et al. [48] reported that FA application enhanced the total phenol and flavonoid content in yarrow flowers, which supports the current results. The positive role of FA in improving the total phenolics has been also reported in several species [98,99].

## 5. Conclusions

The current study is the first to report that GE and FA treatments can improve damask rose growth and production. FA was shown to be more effective than GE. According to the gathered data, the positive effects of GE or FA are attributed to the promotion of growth and branching, which in turn increased the flower output and oil yield by enhancing nutritional status. Furthermore, both BIOs improved the phytochemical profile, which was reflected in the enhanced product quality. This report may offer GE or FA as innovative BIOs for commercial application in damask rose production, and they could also be applied to other aromatic plants. However, further research on such plants is required to support the current report.

## Figures and Tables

**Table 1:** The chemical analysis of the ginger extract.

Component	Value
**Biochemical Profile:**	
Fat (g/100 g DW)	2.64
Protein (g/100 g DW)	3.17
Carbohydrates (g/100 g DW)	22.15
Total carotenoids (mg 100 g^−1^ DW)	37.69
Total phenols (mg 100 g^−1^ DW)	347.58
Flavonoids (g 100 g^−1^ DW)	0.21
Citric acid (mg g^−1^ DW)	0.06
**Nutrient Profile** (mg100 g^−1^ DW):	
Phosphorus	10.52
Calcium	29.18
Potassium	174.68
Iron	4.57
Zinc	0.24
Copper	0.18
Magnesium	4.26
Manganese	0.24
**Organic Acids** (mg g^−1^ DW):	
Oxalic acid	14.37
Tartaric acid	22.68

DW; means dry weight.

**Table 2:** Effects of foliar spray with ginger extract and fulvic acid treatments on flower yield attributes of damask rose plants. Means with different letters within a column are significantly different at *p* ≤ 0.05, according to Duncan’s multiple range test.

Treatments	Flowers/hill	Flower Yield/hill (kg)	Flower Yield/ha (kg)	Blind Shoots/hill^-^
Control	680.25 ± 18.25 e	1.585 ± 0.12 e	3962.46 ± 89.15 e	94.08 ± 3.1 a
GE1	723.24 ± 19.24 d	1.830 ± 0.11 d	4574.49 ± 90.15 d	83.27 ± 3.5 b
GE2	762.68 ± 18.69 c	2.120 ± 0.09 c	5300.63 ± 92.14 c	74.57 ± 2.9 c
GE3	789.85 ± 19.24 b	2.227 ± 0.13 b	5568.44 ± 94.65 b	72.42 ± 3.2 c
FA1	754.96 ± 17.58 c	2.023 ± 0.14 c	5058.23 ± 92.15 c	72.47 ± 2.8 c
FA2	807.25 ± 16.57 a	2.365 ± 0.12 a	5913.11 ± 89.87 a	61.37 ±3.2 d
FA3	815.15 ± 19.68 a	2.421 ± 0.13 a	6052.49 ± 94.12 a	62.68 ± 2.8 d

GE1, GE2, and GE3 are ginger extract levels at 5, 10, and 15 mg L^−1^, respectively; FA1, FA2, and FA3 are fulvic acid concentrations at 1, 3, and 5 g L^−1^, respectively.

**Table 3:** GC-MS analysis of damask rose volatile oil, as affected by foliar spray with ginger extract and fulvic acid treatments, on the volatile oil constituents of damask rose plants.

No.	RI	Compound	Control	GE1	GE2	GE3	FA1	FA2	FA3
			Relative (%)						
1.	1028	α-Pinene	3.48	3.51	3.49	3.50	3.52	3.55	3.53
2.	1131	Sabinene	0.09	0.13	0.15	0.14	0.16	0.19	0.17
3.	1136	β-Pinene	0.61	0.63	0.62	0.65	0.68	0.71	0.69
4.	1167	Myrcene	1.74	1.77	1.79	1.83	1.78	1.82	1.87
5.	1192	Limonene	0.29	0.32	0.33	0.32	0.33	0.32	0.35
6.	1486	Linalool	7.17	7.28	7.32	7.29	6.98	7.29	7.27
7.	1511	*cis*-Rose oxide	0.68	0.70	0.69	0.73	0.72	0.76	0.75
8.	1536	Phenyl ethyl alcohol	2.65	2.67	2.68	2.66	2.64	2.78	2.76
9.	1542	*trans*-Rose oxide	0.68	0.66	0.71	0.73	0.72	0.76	0.77
10.	1554	Terpinen-4-ol	1.19	1.22	1.25	1.24	1.28	1.23	1.24
11.	1572	α-Terpineol	2.33	2.35	2.34	2.39	2.38	2.47	2.48
12.	1581	Nerol	7.58	7.56	7.72	7.75	7.32	7.63	7.59
13.	1611	Neral	0.57	0.56	0.59	0.61	0.59	0.64	0.65
14.	1648	Heptadecane	1.54	1.55	1.56	1.57	1.56	1.58	1.57
15.	1677	1-Heptadecene	0.42	0.44	0.43	0.45	0.43	0.46	0.45
16.	1683	Citronellol	18.89	19.42	19.48	19.50	18.92	19.57	19.55
17.	1695	Geraniol	15.56	15.55	15.59	15.62	15.60	15.67	15.69
18.	1711	Geranial	2.57	2.60	2.59	2.63	2.60	2.65	2.64
19.	1725	Eugenol	1.48	1.53	1.52	1.55	1.53	1.57	1.58
20.	1728	Geranyl acetate	0.78	0.77	0.80	0.81	0.79	0.84	0.82
21.	1733	Methyl eugenol	1.35	1.37	1.36	1.39	1.38	1.45	1.46
22.	1739	β-Caryophyllene	0.82	0.84	0.81	0.85	0.83	0.89	0.90
23.	1743	α-Guaiene	1.38	1.37	1.42	1.40	1.42	1.41	1.45
24.	1747	Germacrene D	0.68	0.66	0.69	0.71	0.69	0.74	0.73
25.	1752	δ-Guaiene	1.17	1.18	1.17	1.22	1.19	1.27	1.25
26.	1777	Nonadecene	2.48	2.47	2.51	2.50	2.52	2.56	2.57
27.	1792	Nonadecane	6.98	6.97	6.99	7.11	6.99	7.25	7.23
28.	1815	1-Eicosane	0.38	0.39	0.42	0.41	0.43	0.49	0.51
29.	1886	Heneicosane	1.18	1.22	1.20	1.25	1.20	1.28	1.26
30.	1978	α-Cadinol	0.14	0.18	0.19	0.16	0.21	0.18	0.22
31.	2118	(2E,6Z)-Farnesol	0.21	0.24	0.22	0.27	0.25	0.28	0.26

GE1, GE2, and GE3 are ginger extract levels at 5, 10, and 15 mg L^−1^, respectively; FA1, FA2, and FA3 are fulvic acid concentrations at 1, 3, and 5 g L^−1^, respectively.

**Table 4:** Effects of foliar spray with ginger extract and fulvic acid treatments on the macronutrients (mg g^−1^ DW) and micronutrients (μg g^−1^ DW) of damask rose plants. The means with different letters within a column are significantly different at *p* ≤ 0.05, according to Duncan’s multiple range test.

Treatments	Macronutrients (mg g^−1^ DW) and Micronutrients (μg g^−1^ DW)					
	N	P	K	Mg	Fe	Zn
Control	16.5 ± 0.46 d	2.29 ± 0.16 e	19.8 ± 0.72 d	3.02 ± 0.12 f	251.25 ± 7.48 e	64.84 ± 2.54 e
GE1	19.8 ± 0.54 c	3.2 ± 0.09 d	21.0 ± 0.63 c	3.84 ± 0.14 e	268.98 ± 8.17 c	71.65 ± 3.15 d
GE2	21.4 ± 0.43 b	3.6 ± 0.08 c	22.2 ± 0.84 b	4.15 ± 0.16 c	275.64 ± 8.68 c	74.57 ± 2.71 c
GE3	21.9 ± 0.35 b	3.7 ± 0. 17 bc	22.4 ± 0.72 b	4.48 ± 0.15 b	286.84 ± 8.12 b	78.95 ± 2.14 ab
FA1	19.7 ± 0.64 c	3.4 ± 0.14 c	21.9 ± 0.57 c	3.96 ± 0.16 d	276.57 ± 7.14 c	73.54 ± 2.45 cd
FA2	22.9 ± 0.42 a	3.9 ± 0.15 ab	23.1 ± 0.68 a	4.69 ± 0.13 a	293.94 ± 8.68 ab	77.65 ± 2.87 b
FA3	23.1 ± 0.51 a	4.1 ± 0.16 a	23.3 ± 0.82 a	4.76 ± 0.14 a	296.48 ± 7.74 a	81.95 ± 2.65 a

GE1, GE2, and GE3 are ginger extract levels at 5, 10, and 15 mg L^−1^, respectively; FA1, FA2, and FA3 are fulvic acid concentrations at 1, 3, and 5 g L^−1^, respectively.

## Data Availability

Not applicable.
